# Logic-based modeling of biological networks with Netflux

**DOI:** 10.1371/journal.pcbi.1012864

**Published:** 2025-04-04

**Authors:** Alexander P. Clark, Mukti Chowkwale, Alexander Paap, Stephen Dang, Jeffrey J. Saucerman

**Affiliations:** 1 Department of Biomedical Engineering, University of Virginia, Charlottesville, Virginia, United States of America; 2 Robert M. Berne Cardiovascular Research Center, University of Virginia, Charlottesville, Virginia, United States of America; SIB Swiss Institute of Bioinformatics, SWITZERLAND

## Abstract

Molecular signaling networks drive a diverse range of cellular decisions, including whether to proliferate, how and when to die, and many processes in between. Such networks often connect hundreds of proteins, genes, and processes. Understanding these complex networks is aided by computational modeling, but these tools require extensive programming knowledge. In this article, we describe a user-friendly, programming-free network simulation tool called Netflux. Over the last decade, Netflux has been used to construct numerous predictive network models that have deepened our understanding of how complex biological networks make cell decisions. Here, we provide a Netflux tutorial that covers how to construct a network model and then simulate network responses to perturbations. Upon completion of this tutorial, you will be able to construct your own model in Netflux and simulate how perturbations to proteins and genes propagate through signaling and gene-regulatory networks.

## 1 Introduction

Biological signaling networks are responsible for a variety of cellular decisions, including growth, proliferation, disease progression, and death. These varied behaviors arise from interactions between hundreds of proteins, genes, and cellular processes. This complexity makes it difficult to understand mechanisms of cellular decisions through reductionist experimental approaches alone. Computational modeling addresses this challenge by integrating known interactions between system components into a framework; one can then use computer simulations to explore biological processes *in silico*, providing a means to rapidly generate testable predictions.

**Fig 1:**
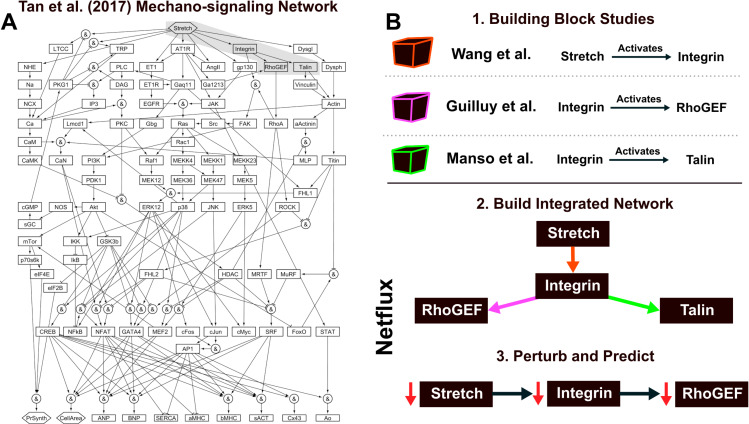
(A) Network model from Tan et al. (2017, [1]) shows how complex networks can be constructed through manual curation. Subsequent simulations in this study provide novel insights into a biological process (in this case, cardiomyocyte mechano-signaling). (B) Individual reductionist studies [2–4] provide connections between network components. These individual connections can be integrated into a network model. Subsequent perturbation studies can make novel predictions of previously untested genetic/protein perturbations—these come with a mechanistic explanation for the perturbation outcome.

Understanding the signaling involved in biological processes can take decades of rigorous, reductionist cause-and-effect research. For example, a connection between high blood pressure and cardiac hypertrophy (often a precursor to heart failure) has been established for years, but until recently [1] (**Fig 1A**), the complex mechano-signaling pathways that drive this remodeling had not been integrated at a systems level. Over decades, individual studies have connected components (e.g., stretch activating integrin) and explained mechano-signaling pathways (e.g., a multi-protein pathway whereby stretch activates Ras) in cardiomyocytes. Collectively, these studies demonstrate the complex role of mechano-signaling in cardiac hypertrophy, but they do not provide a single mechanistic picture of stretch-induced hypertrophy.

Network models offer a means to integrate the high-quality reductionist building block studies mentioned above into a framework to study the complexity of biological signaling processes (**Fig 1B**). Building block studies identify the nature of interactions, like whether stretch increases or decreases the expression of calcium channels—it increases them. By mining many of these reductionist studies, one can create a network of interactions between species and construct an integrated picture of how individual interactions can lead to an emergent phenotype. For example, **Fig 1A** shows a mechano-signaling network for heart cells that was manually curated using data from over 170 studies [1]. This network includes mechanical Stretch as an input that stimulates several pathways consisting of receptors (AT1R, angiotensin type 1 receptor), ion channels (LTCC, L-type calcium channel), transcription factors (GATA4), sarcomeric proteins (bMHC, beta myosin heavy chain), and ions (Ca, Calcium). The interactions can be either activating or inhibiting. For example, the Stretch input signal increases (denoted as solid arrow) the expression of L-type calcium channels. In contrast protein kinase G 1 (PKG1) inhibits the expression of L-type calcium channels. The 125 activating or inhibitory interactions within this network give rise to emergent phenotypic characteristics, like Cell Area.

Coding these individual interactions into a cohesive network and then simulating genetic or pharmacologic perturbations allows us to uncover insights that are not apparent when studying components in isolation. Network modeling can prioritize pathways that work together or in tension to result in emergent phenomena, such as changes in Cell Area. By simulating these networks, researchers can explore how different pathways interact, identify key regulatory nodes, and predict system behavior under various conditions. For example, through systematic perturbation to the mechano-signaling network in [1], the authors identified mechanisms by which increased Stretch can increase Cell Area, a maladaptive change in heart cell physiology. Knowing the mechanism, they showed how a recently approved combination heart failure drug called Entresto attenuates heart failure progression through distinct, yet synergistic, mechano-signaling pathways. This systems approach demonstrates how modeling and simulation can harness the complexity of molecular networks to identify biological mechanisms of disease progression and explain novel therapeutic approaches.

Decades of single-gene cause-effect research provide the building blocks for constructing comprehensive network models. Many such studies include the directionality of relationships (e.g., X activates or inhibits Y) without precise measures of magnitude or kinetic parameters required by traditional mass action modeling approaches. Boolean [5] and fuzzy logic [6] modeling approaches have been used to study qualitative features of feedback loops and cell state transitions. These methods were originally applied to model gene regulatory networks where genes are assumed to be fully on or fully off. A benefit of such approaches is that they do not require precisely measured kinetic parameters; however, they cannot predict the relative quantities of species activity or graded crosstalk between pathways. To address these limitations, the logic-based differential equations used in Netflux instead treat activation and inhibition as continuous relationships [7], which allow for semi-quantitative strengths of protein or gene interactions. While Boolean approaches represent pathway crosstalk with discrete AND and OR gates, logic-based differential equations use continuous AND and OR gates that predict graded crosstalk.

As the amount of biological data continues to grow, and new methods/databases make it easier to find connections between molecules, there is an increased need for tools that facilitate the development and simulation of biological networks. Historically, building and simulating such complex network models required extensive programming experience. Recently, user-friendly tools [8–11] including Netflux have lowered the barrier to entry to modeling of biological networks, each using different modeling approaches with various tradeoffs discussed above. In this manuscript, we provide a detailed introduction to Netflux (https://github.com/saucermanlab/Netflux), a programming-free tool for constructing and simulating biological networks using our above-described logic-based differential equations with normalized Hill equations [7].

Netflux is implemented in MATLAB and. The normalized Hill function describes the steady-state activation or inhibition from one species to another on a continuous scale, using differential equations for reproducible simulations in continuous time. In contrast, traditional Boolean approaches assume that genes or proteins are simply on or off, while still requiring complex and stochastic updating schemes. Netflux performs all of this math “under the hood”, allowing users to construct networks and run simulations without any coding. If you’re interested in learning about how Netflux models are represented mathematically as logic-based differential equations, details can be found in Kraeutler et al. (2010, [7]). The tool has been used by scientists of various levels and technical proficiency to develop models of numerous biological networks that have led to meaningful insights and advances in our understanding of human disease [12–16]. Netflux has also been used as an educational tool at the undergraduate and high school levels to introduce the concepts of network biology.

By the end of this article, you will be able to use Netflux to create a biological network model and then predict how the network responds to perturbations in environmental conditions, proteins, and genes. We will also discuss advanced Netflux topics and provide published examples of how models built with Netflux provide meaningful insights into biological processes.

## 2 Getting started with Netflux

### Installation

Download Netflux from GitHub (https://github.com/saucermanlab/Netflux). It can either be: option 1) installed as a desktop application, or option 2) opened and run directly from MATLAB.

### Loading a Model

**Fig 2:**
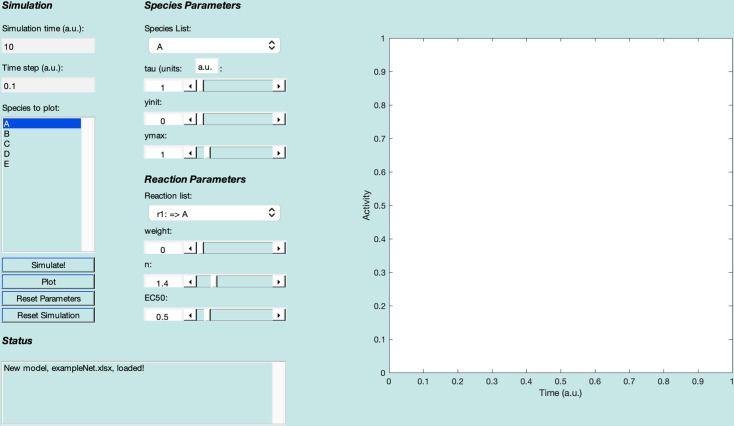
Netflux graphical user interface. The Netflux GUI includes four sections (left) and a plot (right) of species activity over time. (1) ***Simulation*** includes information about the simulation time and species that will be plotted in the panel on the right. (2) ***Status*** includes information on the state of the loaded model and is important to check after loading models and running simulations. (3) ***Species Parameters*** includes information about the species initial value (yinit), maximum value (ymax), and how quickly it can change (tau). (4) ***Reaction Parameters*** includes information about a reaction’s strength (weight), the steepness of activation (n), and the half maximal effective concentration (EC50), which are collectively responsible for producing dynamic changes in species.

Open the Netflux graphical user interface (GUI, Fig 2) by either clicking on the installed application (option 1) or running the “Netflux.m” file within MATLAB (option 2).

### Loading the examplenet model

Netflux includes a repository of published network models in the “models” subfolder. We will first focus on the ExampleNet model [7].

Open the “exampleNet.xlsx” file by selecting Open from the File tab within the Netflux GUI and then navigate to models/exampleNet.xlsx.

**Fig 3:**
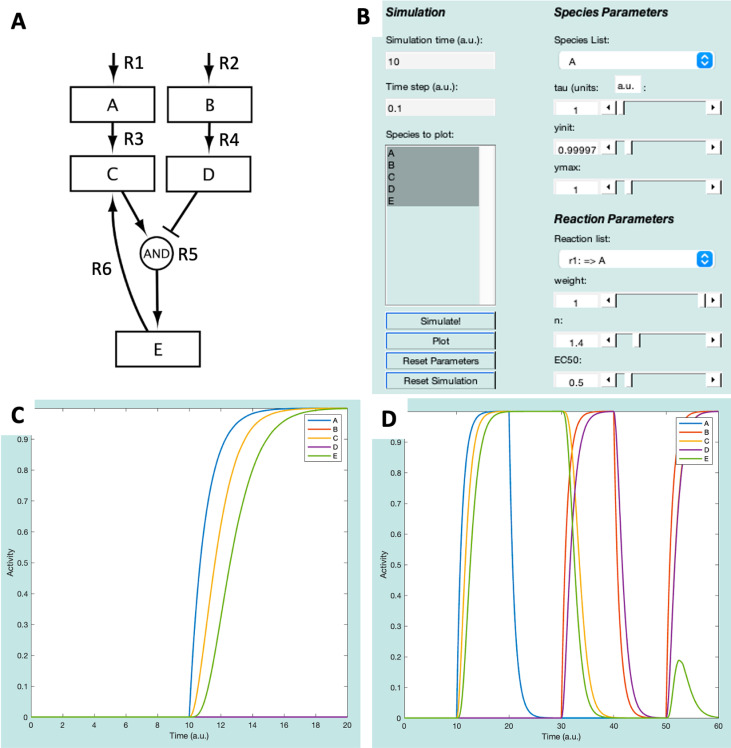
ExampleNet (A) schematic and (B) GUI representation. (C) plot of species after Simulations 1 and 2. (D) Plot of species after all simulations.

In Netflux, we refer to network genes or proteins as *species* and the activating or inhibiting interaction between species as a *reaction*. The ExampleNet model (Fig 3A) consists of five species (A, B, C, D, and E) and six reactions, including two input species (A and B) that feed into the network and can be turned on or off by turning on or off their respective input reactions.

- **(Input) Reaction 1:** Environmental stimulus that activates Species A.
- **(Input) Reaction 2:** Environmental stimulus that activates Species B.
- **Reaction 3:** Species A activates Species C.
- **Reaction 4**: Species B activates Species D.
- **Reaction 5**: Species C activates species E, and species D inhibits species E.
- **Reaction 6:** Species E activates species C.

Once loaded, all the network species are displayed in the “Species to plot” box of the Netflux GUI. For each species or reaction, there is a set of ***Species Parameters*** or ***Reaction Parameters***. These parameters include:

- tau—time constant, describing the rate of change for a given species
- yinit—the initial value, before simulation, for a given species
- ymax—the maximum value for a given species
- weight—the strength of the relationship between two species
- n—the cooperativity of the relationship between two species
- EC50—the half maximal concentration of the interaction

Under ***Species Parameters***, verify that the values for time constants (tau=1), initial values (yinit=0), and maximum values (ymax=1) are the same for each species. Additionally, under ***Reaction Parameters*** (bottom right of the GUI), verify that the reaction weight (weight=0 or 1), Hill coefficient (n=1.4), and half maximal effective concentration (EC50=0.5) are preset for each reaction. These default parameter values have been shown to provide good prediction accuracies, and often work without the need for tuning.

### Simulating ExampleNet in response to environmental stimuli

The model is ready to be run. Netflux uses Matlab’s ode15s solver when running the simulation.

First, select all the species in the “Species to plot” box—this can be done by selecting the A, holding the “Shift” key, and then selecting E (Fig 3B). Follow the steps below and refer to the GUI in Fig 3B to run the model and plot its results:

1) Press the “Simulate!” button. The axes on the right side of the GUI should change, but none of the species should have an Activity above 0.
2) Click the “Reaction list” to see the reactions that are defined in ExampleNet. Change the reaction weight for “r1: => A” to 1, then click “Simulate!” again. Now, between timepoints 10 and 20, species A, C, and E should all increase to an Activity level of 1 (Fig 3C).
3) Simulate washout of A by changing the “r1: => A” reaction weight back to 0 and then clicking “Simulate!”.
4) Simulate stimulation with B by changing the “r2: => B” reaction weight to 1 and then clicking “Simulate!”.
5) Simulate washout of B by changing the “r2: => B” reaction weight back to 0 and then clicking “Simulate!”.
6) Finally, simulate simultaneous stimulation with both A and B by changing their input reaction weights to 1 and then clicking “Simulate!”.

Execution of these six steps will result in the plot displayed in Fig 3D. Before starting a new simulation, select “Reset Parameters” and “Reset Simulation”.

## 3 Build and simulate a new network model

**Fig 4:**
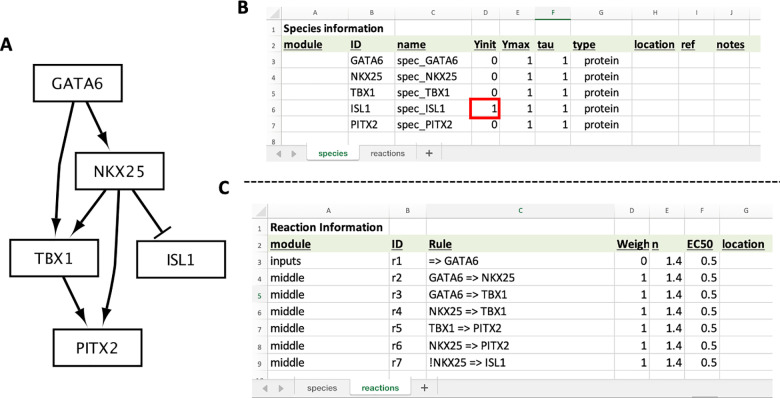
Model of a cardiac development gene regulatory network. (**A**) CardiacDevNet schematic. (**B**) Excel sheets for the CardiacDevNet Species and (**C**) Reaction information. Notice, the ISL1 Yinit value starts at 1 because it is inhibited by NKX25, which has a Yinit of 0 (**B**, red box).

This section will cover how to build a new model from scratch. The model used in this section is part of a recently elucidated gene regulatory network that is essential for proper heart formation [17], visualized in Fig 4A. In addition to model construction, we will simulate the knockout of a gene within this network and compare the results to experimental data linking dysregulation of this network to congenital heart defects.

### Creating the CardiacDevNet model

First, make a copy of the “exampleNet.xlsx” model in Netflux/models/, and rename it “cardiacDevNet.xlsx”. Note, when building a new model, it is advised to follow the format of a previously developed model (e.g., “exampleNet.xlsx”).

### Adding the model species

This model includes five transcription factor species, with the following known actions (within this biological context):

- GATA6—shown to bind an enhancer of NKX2-5 that is accessible in a special subset of cardiac progenitor cells found in the anterior second heart field.
- NKX2-5—well-characterized protein that regulates several genes essential for proper heart formation.
- TBX1—well-characterized protein that regulates several genes essential for proper heart formation.
- ISL1—plays a role in forming the cardiac progenitor pool but its repression is important for cells to continue differentiation towards adult cardiomyocytes.
- PITX2—known to regulate formation of a region of the heart called the outflow tract.

Open “cardiacDevNet.xlsx” in Excel or Google Sheets and navigate to the “species” tab. Edit the ID, name, and Yinit fields to match Fig 4A and4B. Notice, the Yinit value for ISL1 should start at 1. This is because the subset of specialized cardiac progenitor cells (the anterior second heart field cells) starts with high levels of ISL1.

### Adding the model reactions

This model has the following seven reactions (Fig 4A and,4C), including one input reaction:

- **(Input) Reaction 1:** GATA6 is expressed. This is modeling the expression of GATA6 that is present within this specialized subset of cardiac progenitor cells.
- **Reaction 2:** GATA6 activates NKX2-5.
- **Reaction 3**: GATA6 activates TBX1.
- **Reaction 4**: NKX2-5 activates TBX1.
- **Reaction 5:** TBX1 activates PITX2.
- **Reaction 6:** NKX2-5 activates PITX2.
- **Reaction 7:** NKX2-5 inhibits the expression of ISL1. In Excel, an exclamation mark is used to denote that NKX2-5 inhibits ISL1 (Fig 4C, ID=r7).

Navigate to the “reactions” tab of “cardiacDevNet.xlsx”. Edit the module, ID, Rule, and Weight to match Fig 4C. For the “r1” reaction rule (“=> GATA6”), include a single quote before typing the equals character (=) to ensure Excel properly interprets the coded interaction.

### Simulating normal development with CardiacDevNet

Save “cardiacDevNet.xlsx” in Excel or export it from Google Sheets as a.xlsx file. Next, open Netflux by either clicking on the installed application or running the “Netflux.m” file within MATLAB and then open “cardiacDevNet.xlsx” (see Section 2 for details).

**Fig 5:**
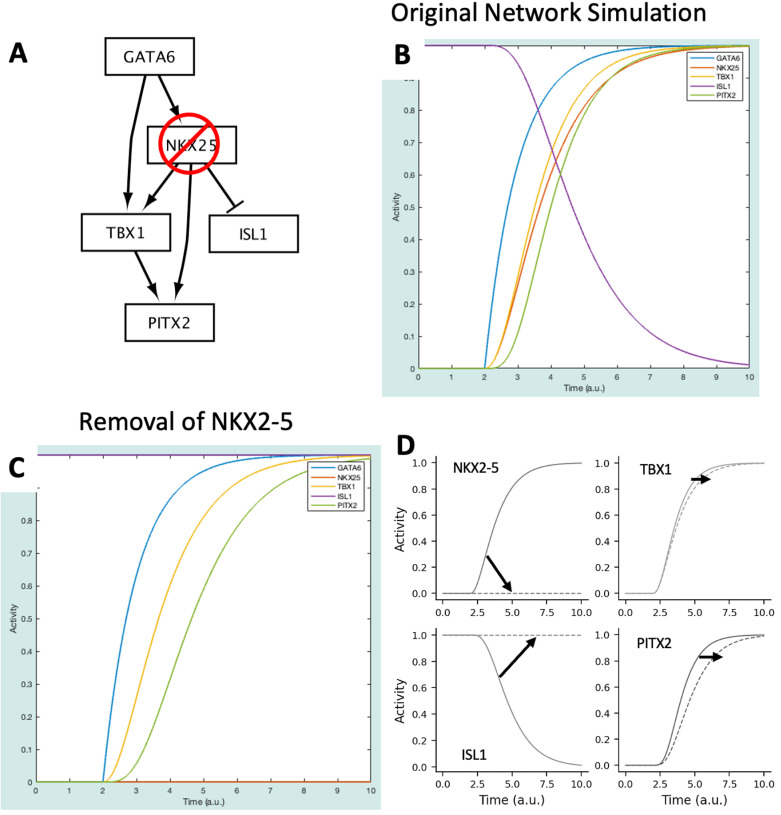
(A) CardiacDevNet NKX2-5 knockout schematic. (B), CardiacDevNet simulation results at baseline. (C) CardiacDevNet simulation results after knockout of NKX2-5. (D) Change in simulation results at baseline (solid lines) and after NKX2-5 knockout (dashed lines) for the transcription factors: NKX2-5, TBX1, ISL1, and PITX2.

The following steps cover how to run a simulation of the normal cardiac development:

1. Set the simulation time to 2. This step improves visualization of results in this simulation. It is not always needed.
2. Select all species to plot. This is done by clicking on GATA6, holding the “Shift” key, and then selecting PITX2.
3. Select the “Simulate!” button. This should change the axes, but there should be no change in species Activity.
4. Change the simulation time to 8.
5. Select “r1: => GATA6” from the “Reaction list” dropdown and change the weight to 1.
6. Select the “Simulate!” button. The results should match the solid lines plotted in Fig 5B.

This simulation shows that NKX2-5, TBX1, and PITX2 increase while ISL1 decreases in response to an increase in GATA6 (Fig 5B, solid lines). These gene expression dynamics are important for anterior second heart field cells to progress towards mature outflow tract cardiomyocytes.

### Simulating a gene knockout with CardiacDevNet

In [17], authors reduced the expression of NKX2-5—here, we simulate this as a knockout of the NKX2-5 gene. The next steps discuss how to conduct an *in silico* gene knockout study using the model (results displayed in Fig 5C):

1. Select “Reset Parameters” and “Reset Simulation”.
2. Select “NKX25” from the “Species List” dropdown and change the ymax to 0. This change simulates the knockout of NKX2-5.
3. Set the Simulation time to 2.
4. Select the “Simulate!” button.
5. Select “r1: => GATA6” from the “Reaction list” dropdown and change the weight to 1.
6. Set the Simulation time to 8.
7. Select the “Simulate!” button.

This simulation represents the conditions under which NKX2-5 is knocked out—this was done by removing NKX2-5 from the network (Fig 5). Taken together, the results of the baseline and perturbation simulations predict the following effects of NKX2-5 knockout (Fig 5D), which largely agrees with the experiments in [17].

1. NKX2-5 is not expressed. This should be interpreted as a reduction in NKX2-5 expression compared to wildtype. In [17], while still present, NKX2-5 expression is significantly reduced by the removal of an enhancer region that, when bound by the GATA6 transcription factor, increases NKX2-5 expression.
2. ISL1 expression is not suppressed. This again agrees with the significant increase in ISL1 observed when NKX2-5 expression is reduced.
3. There is a lag in TBX1 and PITX2 expression. This can be interpreted as a reduction in expression, in agreement with [17], where TBX1 and PITX2 expression was reduced due to a reduction in NKX2-5.

## 4 Discussion, advanced methods and usage

Our simulations with CardiacDevNet illustrate the ease of developing a network model that accurately predicts the outcomes of real-world experiments. Beyond small models such as CardiacDevNet, Netflux has been used to construct networks with tens to 100s of proteins, genes, and reactions. Large-scale network models have been developed with Netflux including those for cardiomyocyte hypertrophy [12,18] and apoptosis [13], fibroblasts [15], macrophages [19], brain endothelial cells [16], mechano-signaling [1], virtual drug screening [20,21], cardiomyocyte differentiation [22], and smooth muscle cells [14]. Several groups have incorporated Netflux-constructed networks into multiscale models to simulate physiology at various spatial scales, including microvascular patterning [23], geometry and composition of the aorta [24], intercellular crosstalk between macrophages and endothelial cells [25], the coupling of contractile mechanics to chemical signaling in vascular smooth muscle cells [26], and coupling hormonal signaling and organ-scale biomechanics that drive how the heart remodels during pregnancy [27]. As networks increase in size and complexity in this way, there are a few additional considerations to be made.

*Network Visualization*: Netflux provides a tool to export networks in a format that can be loaded into Cytoscape [28], a user-friendly software for network visualization. For example, Cytoscape was used to produce the visualizations in Figs 2–4.

*Citation Tracking:* The Netflux-formatted.xlsx sheets include several columns within the “species” and “reactions” tabs that can be used to keep track of references and metadata. This becomes increasingly important as models grow large and incorporate insights from numerous independent studies.

*Model Exporting:* Netflux also provides functionality for exporting models to MATLAB or Python, making it possible to run more sophisticated analyses and conduct large-scale, systematic perturbation studies.

*Model Validation*: As noted above, network models often draw on many different independent, biologically targeted studies to define the network structure—this provides confidence in model definition. However, experimental validation is critical to build confidence in model predictions. Experimental validation is performed using *in vitro* or *in vivo* perturbation experiments from the literature that were not used to develop the model. Setting aside specific validation experiments that are distinct from those used to develop the model is essential. Typically, the validation dataset involves experiments where perturbations induce increases or decreases in specific network species. These qualitative changes are then compared to the qualitative predictions made by the model. Across a large number of studies, logic-based differential equation models can achieve validation accuracies exceeding 70% [29–32], providing strong evidence for the reliability of their predictions. Subsequently, the model can be used to test model predictions under previously untested conditions. Whenever possible, it is strongly recommended to carry out these experiments and compare the results to the model.

*Parameter estimation and uncertainty quantification:* While not in Netflux itself, the logic-based differential equation code generated by Netflux enables tuning of the parameters with quantitative data to further increase model accuracy [12,14]. While these approaches can improve model utility, such flexibility increases the risk of misuse of the model if assumptions and limitations are not well understood. For example, overfitting a model or failing to validate predictions experimentally can undermine its biological relevance. To mitigate these risks, assumptions must be clearly documented, iterative rounds of model validation should be performed, and results interpreted in the context of their biological system, ideally through new experiments. These steps increase confidence that the model will generalize to make meaningful, novel predictions.

The precise parameter values for most interactions in these networks are not known. Uncertainty quantification and parameter sensitivity approaches assist in identifying statistical estimates of model outputs when parameters, like the reaction weights in Netflux networks, are unknown. Uncertainty quantification can assess the impact of uncertainty in parameter values, model logic and experimental results used for validation. It can reveal potential sources of bias such as the higher likelihood of certain types of errors in under-powered biological experiments. Methods such as Monte Carlo simulations [33] and polynomial chaos expansions [34] are tools for exploring the effects of parameter and data uncertainty. These tools enable the identification of key parameters, subnetworks, and data limitations that should be prioritized for further investigation to enhance model accuracy.

## 5 Conclusion

Netflux is an accessible tool for constructing and simulating models of biological networks, requiring no programming experience. In this article, we covered the core features of Netflux, including model construction, simulation, perturbation, and were able to produce *in silico* results in agreement with experimental data. While the models used in this article are small compared to networks from many published studies, the concepts remain the same. This article (along with information in the GitHub repository) provides all the information needed to construct your own model and simulate real experiments. As you embark on building network models, we encourage you to rigorously validate your model throughout construction. Well-validated models developed in Netflux can provide both conceptual understanding of biological networks and specific mechanistic predictions of how your network may respond to new experimental perturbations.

## References

[1] Tan PM, Buchholz KS, Omens JH, McCulloch AD, Saucerman JJ. Predictive model identifies key network regulators of cardiomyocyte mechano-signaling. PLoS Comput Biol. 2017;13(11):e1005854. doi: 10.1371/journal.pcbi.1005854 29131824 PMC5703578

[2] Wang N, Butler JP, Ingber DE. Mechanotransduction across the cell surface and through the cytoskeleton. Science. 1993;260(5111):1124–7. doi: 10.1126/science.7684161 7684161

[3] Guilluy C, Swaminathan V, Garcia-Mata R, O’Brien ET, Superfine R, Burridge K. The Rho GEFs LARG and GEF-H1 regulate the mechanical response to force on integrins. Nat Cell Biol. 2011;13(6):722–7. doi: 10.1038/ncb2254 21572419 PMC3107386

[4] Manso AM, Li R, Monkley SJ, Cruz NM, Ong S, Lao DH, et al. Talin1 has unique expression versus talin 2 in the heart and modifies the hypertrophic response to pressure overload. J Biol Chem. 2013;288(6):4252–64. doi: 10.1074/jbc.M112.427484 23266827 PMC3567677

[5] Albert I, Thakar J, Li S, Zhang R, Albert R. Boolean network simulations for life scientists. Source Code Biol Med. 2008;3:16. doi: 10.1186/1751-0473-3-16 19014577 PMC2603008

[6] Aldridge BB, Saez-Rodriguez J, Muhlich JL, Sorger PK, Lauffenburger DA. Fuzzy logic analysis of kinase pathway crosstalk in TNF/EGF/insulin-induced signaling. PLoS Comput Biol. 2009;5(4):e1000340. doi: 10.1371/journal.pcbi.1000340 19343194 PMC2663056

[7] Kraeutler MJ, Soltis AR, Saucerman JJ. Modeling cardiac β-adrenergic signaling with normalized-Hill differential equations: comparison with a biochemical model. BMC Syst Biol. 2010;4:157. doi: 10.1186/1752-0509-4-157 21087478 PMC2993667

[8] Helikar T, Kowal B, McClenathan S, Bruckner M, Rowley T, Madrahimov A, et al. The cell collective: toward an open and collaborative approach to systems biology. BMC Syst Biol. 2012;6:96. doi: 10.1186/1752-0509-6-96 22871178 PMC3443426

[9] Hoops S, Sahle S, Gauges R, Lee C, Pahle J, Simus N, et al. COPASI—a COmplex PAthway SImulator. Bioinformatics. 2006;22(24):3067–74. doi: 10.1093/bioinformatics/btl485 17032683

[10] Cowan AE, Moraru II, Schaff JC, Slepchenko BM, Loew LM. Spatial modeling of cell signaling networks. Methods Cell Biol. 2012;110:195–221. doi: 10.1016/B978-0-12-388403-9.00008-4 22482950 PMC3519356

[11] Naldi A, Berenguier D, Fauré A, Lopez F, Thieffry D, Chaouiya C. Logical modelling of regulatory networks with GINsim 2.3. Biosystems. 2009;97(2):134–9. doi: 10.1016/j.biosystems.2009.04.008 19426782

[12] Khalilimeybodi A, Paap AM, Christiansen SLM, Saucerman JJ. Context-specific network modeling identifies new crosstalk in β-adrenergic cardiac hypertrophy. PLoS Comput Biol. 2020;16(12):e1008490. doi: 10.1371/journal.pcbi.1008490 33338038 PMC7781532

[13] Grabowska ME, Chun B, Moya R, Saucerman JJ. Computational model of cardiomyocyte apoptosis identifies mechanisms of tyrosine kinase inhibitor-induced cardiotoxicity. J Mol Cell Cardiol. 2021;155:66–77. doi: 10.1016/j.yjmcc.2021.02.014 33667419 PMC8154673

[14] Estrada AC, Irons L, Rego BV, Li G, Tellides G, Humphrey JD. Roles of mTOR in thoracic aortopathy understood by complex intracellular signaling interactions. PLoS Comput Biol. 2021;17(12):e1009683. doi: 10.1371/journal.pcbi.1009683 34898595 PMC8700007

[15] Zeigler AC, Richardson WJ, Holmes JW, Saucerman JJ. A computational model of cardiac fibroblast signaling predicts context-dependent drivers of myofibroblast differentiation. J Mol Cell Cardiol. 2016;94:72–81. doi: 10.1016/j.yjmcc.2016.03.008 27017945 PMC4861657

[16] Gorick CM, Saucerman JJ, Price RJ. Computational model of brain endothelial cell signaling pathways predicts therapeutic targets for cerebral pathologies. J Mol Cell Cardiol. 2022;164:17–28. doi: 10.1016/j.yjmcc.2021.11.005 34798125 PMC8958390

[17] Yamaguchi N, Chang EW, Lin Z, Shekhar A, Bu L, Khodadadi-Jamayran A, et al. An anterior second heart field enhancer regulates the gene regulatory network of the cardiac outflow tract. Circulation. 2023;148(21):1705–22. doi: 10.1161/CIRCULATIONAHA.123.065700 37772400 PMC10905423

[18] Ryall KA, Holland DO, Delaney KA, Kraeutler MJ, Parker AJ, Saucerman JJ. Network reconstruction and systems analysis of cardiac myocyte hypertrophy signaling. J Biol Chem. 2012;287(50):42259–68. doi: 10.1074/jbc.M112.382937 23091058 PMC3516769

[19] Liu X, Zhang J, Zeigler AC, Nelson AR, Lindsey ML, Saucerman JJ. Network analysis reveals a distinct axis of macrophage activation in response to conflicting inflammatory cues. J Immunol. 2021;206(4):883–91. doi: 10.4049/jimmunol.1901444 33408259 PMC7854506

[20] Zeigler AC, Nelson AR, Chandrabhatla AS, Brazhkina O, Holmes JW, Saucerman JJ. Computational model predicts paracrine and intracellular drivers of fibroblast phenotype after myocardial infarction. Matrix Biol. 2020;91–92:136–51. doi: 10.1016/j.matbio.2020.03.007 32209358 PMC7434705

[21] Rogers JD, Aguado BA, Watts KM, Anseth KS, Richardson WJ. Network modeling predicts personalized gene expression and drug responses in valve myofibroblasts cultured with patient sera. Proc Natl Acad Sci U S A. 2022;119(8):e2117323119. doi: 10.1073/pnas.2117323119 35181609 PMC8872767

[22] Hota SK, Rao KS, Blair AP, Khalilimeybodi A, Hu KM, Thomas R, et al. Brahma safeguards canalization of cardiac mesoderm differentiation. Nature. 2022;602(7895):129–34. doi: 10.1038/s41586-021-04336-y 35082446 PMC9196993

[23] Leonard-Duke J, Agro SMJ, Csordas DJ, Bruce AC, Eggertsen TG, Tavakol TN, et al. Multiscale computational model predicts how environmental changes and drug treatments affect microvascular remodeling in fibrotic disease. bioRxiv. 2024;2024.03.15.585249. doi: 10.1101/2024.03.15.585249 39720203 PMC11667245

[24] Estrada AC, Irons L, Tellides G, Humphrey JD. Multiscale computational model of aortic remodeling following postnatal disruption of TGFβ signaling. J Biomech. 2024;169:112152. doi: 10.1016/j.jbiomech.2024.112152 38763809 PMC11141772

[25] Patidar K, Versypt ANF. Logic-based modeling of inflammatory macrophage crosstalk with glomerular endothelial cells in diabetic kidney disease. bioRxiv. 2024;2023.04.04.535594. doi: 10.1101/2023.04.04.535594 37066138 PMC10104015

[26] Flanary SM, Barocas VH. A structural bio-chemo-mechanical model for vascular smooth muscle cell traction force microscopy. Biomech Model Mechanobiol. 2023;22(4):1221–38. doi: 10.1007/s10237-023-01713-6 37004657 PMC10603623

[27] Yoshida K, Saucerman JJ, Holmes JW. Multiscale model of heart growth during pregnancy: integrating mechanical and hormonal signaling. Biomech Model Mechanobiol. 2022;21(4):1267–83. doi: 10.1007/s10237-022-01589-y 35668305

[28] Shannon P, Markiel A, Ozier O, Baliga NS, Wang JT, Ramage D, et al. Cytoscape: a software environment for integrated models of biomolecular interaction networks. Genome Res. 2003;13(11):2498–504. doi: 10.1101/gr.1239303 14597658 PMC403769

[29] Fowler A, Knaus KR, Khuu S, Khalilimeybodi A, Schenk S, Ward SR, et al. Network model of skeletal muscle cell signalling predicts differential responses to endurance and resistance exercise training. Exp Physiol. 2024;109(6):939–55. doi: 10.1113/EP091712 38643471 PMC11140181

[30] Khalilimeybodi A, Riaz M, Campbell SG, Omens JH, McCulloch AD, Qyang Y, et al. Signaling network model of cardiomyocyte morphological changes in familial cardiomyopathy. J Mol Cell Cardiol. 2023;174:1–14. doi: 10.1016/j.yjmcc.2022.10.006 36370475 PMC10230857

[31] Irons L, Humphrey JD. Cell signaling model for arterial mechanobiology. PLoS Comput Biol. 2020;16(8):e1008161. doi: 10.1371/journal.pcbi.1008161 32834001 PMC7470387

[32] Frank DU, Sutcliffe MD, Saucerman JJ. Network-based predictions of in vivo cardiac hypertrophy. J Mol Cell Cardiol. 2018;121:180–9. doi: 10.1016/j.yjmcc.2018.07.243 30030017 PMC6175293

[33] Fox BL. Strategies for Quasi-Monte Carlo. Boston, MA: Springer; 1999. doi: 10.1007/978-1-4615-5221-5

[34] Xiu D, Karniadakis GE. The Wiener--Askey Polynomial Chaos for Stochastic Differential Equations. SIAM J Sci Comput. 2002;24(2):619–44. doi: 10.1137/s1064827501387826

